# Sociologic Studies of a Medico-Legal Nature

**Published:** 1903-06

**Authors:** 


### Books and Magazines.

---

SOCIOLOGIC STUDIES OF A MEDICO-LEGAL NATURE, by Louis J. Rosenberg, LL. B., Associate of the Victorian Institute, London, etc., N. E. Oranstam, M. D., Ph. G., assistant in Chemistry and Dermatology, Michigan College of Medicine and Surgery, etc., Detroit, with introduction by Hon. Clark Bell, LL. D., President of the Medico-Legal Society. 137 pages; price $1.00. Chicago, P. G. Englehard & Co. 1902.

Much that appears in this book first came out in the *Medico-Legal Journal.* Several of the articles read well, but they seem to lack point and arrive at nothing but generalizations. Tolstoi appears to be the character imitated, but one gets little for his time out of the book. T. J. B.

